# Thermal performance evaluation of bio-bricks and conventional bricks in residential buildings in Aswan city, Egypt

**DOI:** 10.1038/s41598-023-42228-5

**Published:** 2023-09-25

**Authors:** Rania Emad Abd El-Hady, Abdelaziz Farouk A. Mohamed

**Affiliations:** 1 Architectural Engineering and Environmental Design Department, Arab Academy for Science, Technology & Maritime Transport (South Valley Branch), El Sadadt Street, Aswan City, Egypt; 2 Architectural Engineering and Environmental Design Department, Arab Academy for Science, Technology & Maritime Transport, Heliopolis, Egypt

**Keywords:** Engineering, Materials science

## Abstract

From raw material extraction to final product disposal, the construction industry is integrally involved in every stage of the greenhouse gas emissions life cycle. One of the main causes of the climate catastrophe is the increasing use of polluting energy sources to power our homes and businesses. This massive problem of global warming has now forced countries to act. To further address sustainability, they seek to reduce energy consumption and CO_2_ emissions by adopting more sustainable materials. The current trend in scientific research is to use waste resources to improve the properties of various materials to exacerbate the problems of climate change because of the use of traditional building materials. Therefore, one of the most environmentally friendly alternatives to the standard procedure is the use of agricultural residues to improve the quality of building materials. This improvement will modify the thermal properties of building materials such as bricks, which will lead to an improvement in energy efficiency inside buildings, especially residential buildings. As a result, the research focused solely on simulating several bio-brick alternatives that had been discovered in earlier studies in order to test their viability in terms of increasing the energy efficiency of residential buildings in one of the hot cities. The study demonstrated that using bio-building materials can lower energy usage. In addition to saving energy in residential constructions, rice straw cement bricks and sugarcane bricks have operating efficiency rates of roughly 7% and 12%, respectively. All these advancements over conventional brick reduce greenhouse gas emissions and carbon dioxide.

## Introduction

The concentration of atmospheric greenhouse gases (GHGs) is being amplified by human activity. With the continuous increase in the world's population, the problems related to using raw materials increased, leading to an environmental imbalance. However, in recent years, environmental issues have not been reduced to this, but awareness has increased. As a result, the extent of the built environment's impact on environmental problems is known^1^ . So, Nowadays, countries care about the global warming problem and the greenhouse effect, which acts actively on our plants. So now researchers study reasons for environmental pollution and greenhouse effects to face risks (climate change, urban heat islands, etc.). Globally, buildings play a crucial role in the use of energy. The construction industry considerably impacts overall natural resource usage and emissions^2,3^. The increase in waste creation is a consequence of population growth, which has a direct impact on both the environment and the economy. In recent times, agricultural residues have emerged as a notable contributor to environmental contamination. The indiscriminate incineration of agricultural waste, such as straw and livestock manure, within the context of an agrarian nation, has resulted in a multitude of environmental issues. The escalating volume of garbage and its improper disposal, particularly in developing nations, have consistently posed a significant threat to the safety of the environment and the health of its inhabitants. Additionally, this issue has exacerbated the contribution of these countries to the global emission of greenhouse gases^4^. Since the beginning of the twentieth century, Construction has become entirely dependent on concrete structures and fired clay bricks, whose manufacture leads to the emission of enormous quantities of greenhouse gases. Moreover, despite their high compressive properties, their thermal properties are poor, leading to an increase in microclimate problems. The operational energy reduction is achieved by substantially increasing the insulating materials. So, a possible strategy to counterbalance this effect is to select building materials with low embodied energy; in this respect, natural materials are perfect candidates because they usually undergo few industrial manufacturing operations, accumulating low embodied energy^1^ . So, many researchers began creating environmental ways to eliminate these wastes by integrating them into different sectors, whether in the paper industry, the building materials industry, animal feeding, or other fields.

### Problem statement

One of the biggest reasons for greenhouse gas emissions is buildings, as buildings are responsible for more than 35% of total emissions because of the energy consumption used in the materials manufacturing phase and the operation phase to achieve thermal comfort for users in different types of buildings.

Conventional building materials used traditionally in the construction phase allow heat to transfer through them, which raises the value of the inside temperature more than the default^5^. 

### Research aim

This research aims to compare conventional materials and biomaterials (bricks) to achieve energy savings in residential buildings by enhancing building materials (bricks) characteristics, mainly thermal properties, to achieve thermal comfort for and improve human health for residents and decrease active solutions like using air conditioning.

### Research methodology

In this paper, the research depends on the following steps to achieve a comparison between the different types of building materials (traditional and biomaterials), especially for the brick material, and it appeared in:

- Evaluate the functionality of biomaterials in different examples.
- Evaluation of the climatic region of Aswan City as a case study area.
- Evaluate the energy consumption of Aswan city residential buildings, which use conventional materials in construction projects.
- Evaluate the performance if biomaterial (Brick) is used in residential buildings by recycling two different types of agricultural waste available in Upper Egypt.
- Determine the amount of energy savings achieved by replacing conventional brick with bio-brick in residential buildings.
- Studying the different economical alternatives and calculating the life cycle assessment to see the impact of using bio-bricks in saving the cost of the electricity used.

## Bio-building materials properties

Due to their durability and adaptability, bio-based materials are considered valuable construction resources in the twenty-first century. They can be manufactured locally and sustainably, with little shipping expenditure. Moreover, it has been found that this biomaterial is an excellent replacement for traditional materials because it reduces carbon and energy emissions and offers thermal comfort with reduced energy consumption for the operation of buildings^6,7^. 

Several researchers have combined waste materials such as organic waste, waste treatment sludge, fly ash, cigarette butts, rice husk, and processed waste tea into a fired clay brick. This application suggests a way to use waste materials with little environmental impact^8–11^. Researchers in Sri Lanka discovered brick with new properties by adding cow dung ash to the mixture to decrease manufacturing costs and make it a more durable and eco-friendly clay brick. The best ratio for adding waste was 10%, which improved the properties of conventional brick as shown (density from 1450 to 1447 kg/m^3^, water absorption from 12 to 14.5%, and thermal conductivity from 0.85 to 0.2 W/m.k)^12^. 

## Environmental challenges in Egypt

The increase in greenhouse emissions in the world and Egypt resulted in climate change and a marked rise in temperatures, and as a result, the climate affected human health in three ways: a direct effect through weather variables such as heat and storms; and indirectly through natural systems such as disease vectors and pathways caused by human systems such as undernutrition^13,14^. One of the effects of high temperatures is the deaths, injuries, and psychological trauma to which humans are exposed because of extreme natural phenomena, as well as increased respiratory infections, diarrhea, and vascular and heart diseases^14^. Also, due to the environmental deterioration that has been reached, the road has paved the way for viral epidemics transmitted from animal sources^15^. 

The construction sector is one of the critical sectors in the economy of any country, whether it is developed or developing. Therefore, this sector is considered to have an important and prominent position in developing countries' national economies. It undertakes the implementation and establishment of all industrial, agricultural, tourism, infrastructure, and public utility projects and other projects necessary for the comprehensive development of these countries. It is known that the construction industry in Egypt is ancient^16^. 

### Energy efficiency in Egypt

**Figure 1:**
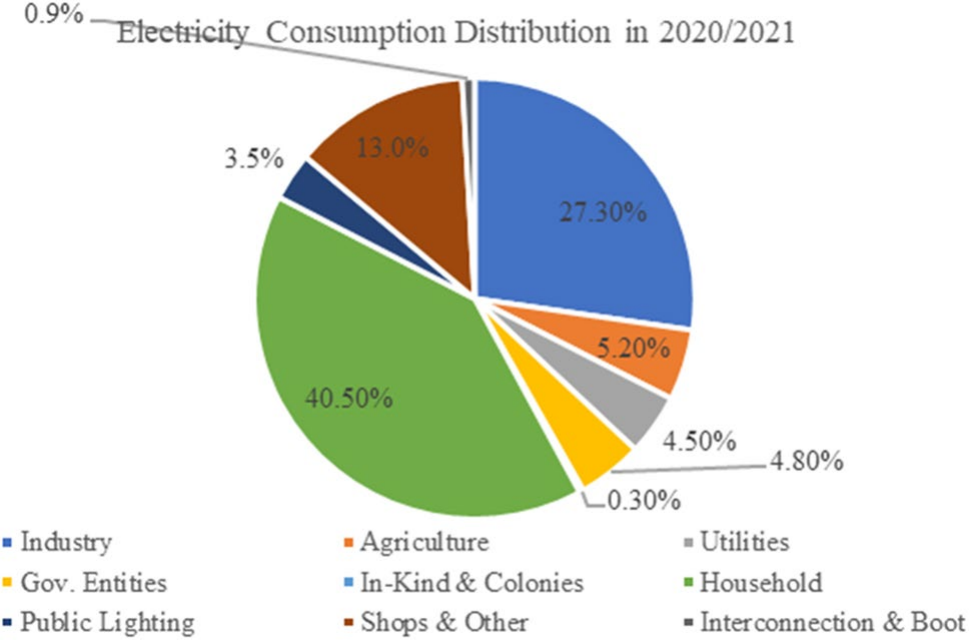
Sold energy according to purposes (2020/2021).

According to the latest report of the Electricity Holding Company for 2020/2021, Electricity consumed in distribution is 40.5% inside residential buildings, 13% in commercial buildings, 4.8% in government buildings, 27.3% in industry, 5.2% in agriculture, and 9.2% in other buildings as shown in Fig. 1. This percentage is a warning sign, as half of the energy consumption is used in residential buildings, which does not result in any applicable product, as in the industrial sector^17^.

### Construction sector in Egypt

**Figure 2:**
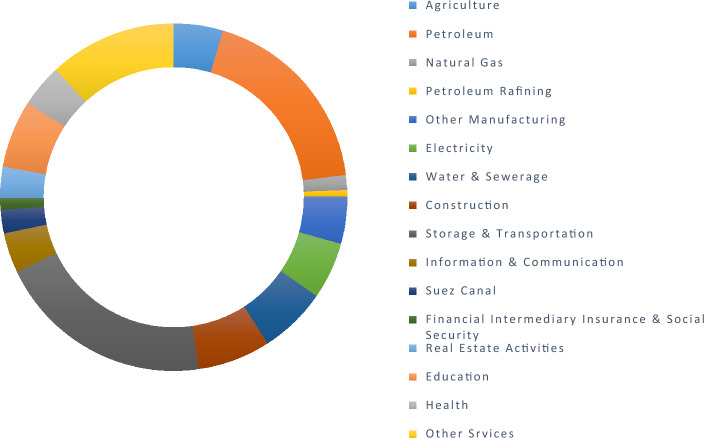
Distribution of public investments for different sectors of the economy in Egypt in 2021/2020.

The construction sector in Egypt is one of the largest and most dynamic sectors in the national economy. It is essential to economic growth through sector relationships, development plans, and various economic activities. Since 2016, the Egyptian government has paid attention to the construction sector since the launch of the economic reform program until the construction sector contributes to the growth of real GDP from 6.3% for the year 2018–2019 to 6.8% for 2020–2021. It is expected that the amount of its contribution to economic growth will increase by 8.5% for the years 2021–2022 as shown in Fig. 2^18^.

**Figure 3:**
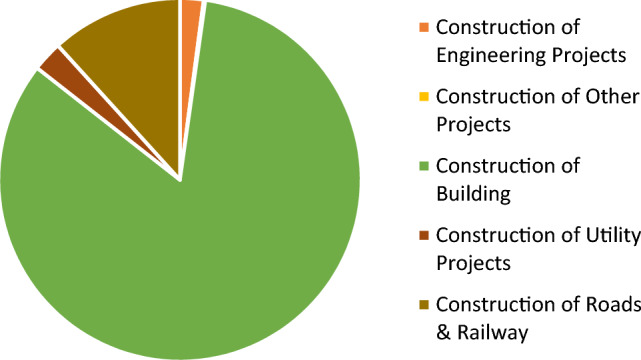
Distributed for construction section of economic activity for 2018/2019.

According to the annual report of the Mobilization and Statistics Authority for the year 2018/2019, the number of projects that have been completed in the construction sector equals 38,830 million pounds, and building projects represent about 83% of the total completed projects as shown in Fig. 3^19^.

According to the interest of the Egyptian government in the construction sector through increasing national projects, the construction sector has become one of the most influential industrial sectors in recent years^20^. 

Egypt enjoys many mineral resources, which are the raw materials for building materials, contributing significantly to the Egyptian economy. They are found in large quantities on the upper surface of the Earth's layers, making their extraction and exploitation easy^20^. 

### Agriculture problems in Egypt

According to the report of the Mobilization and Statistics Authority for the year 2018/2019, the total production of field crops reached 118,486,134 tons, and it included many types of crops, including wheat, barley, corn, rice, legumes, sugar cane, fodder, cotton, and others. According to Omyma Swan, each ton of crop yield generates from 5 to 6 tons of waste, considered an untapped revolution^21^ . Agriculture in Egypt produces annually 33.477 million tons. However, more than 50% are not used. which can be exploited in several industries^21,22^. 

## Case study: applying bio-brick in residential building in Aswan Egypt

**Figure 4:**
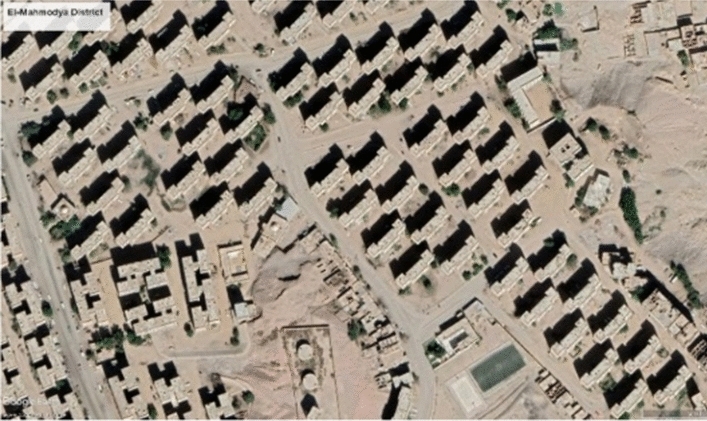
Case-study area google earth map (Google Earth Pro).

**Figure 5:**
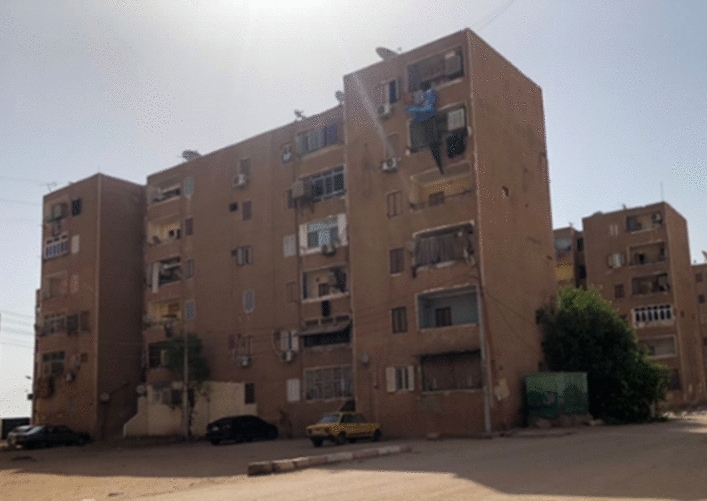
One of block design in existing case (Taken by Authors).

The case study was selected in Aswan (a hot, arid region) to test the effect of modified bricks on thermal comfort in a residential building by reducing the heat gain from walls. The per capita share of green lands is about 0.7 m^2^/person and 5.3 m^2^/person, including the agricultural area north of Aswan city^23^. The case study shows the district covers (0.12 km^2^) area, with built up about 40% of the total area as shown in Fig. 4, and the average building height is 18 m. All structures were constructed using traditional materials as shown in Fig. 5.

On the warmest day in Aswan, which occurred on June 5, the climate of the region under investigation was determined. The yearly maximum and lowest temperatures are 47 °C and 29.5 °C, respectively. To evaluate the impact of bio-bricks in thermal insulation in a hot, arid climate that helped in achieving thermal comfort inside residential buildings without using mechanical facilities, the Onset-HOBO-MX1104 (Analogue/e/Temp/R.H./Light Data Logger) was used daily for 3 months (July, August, and September) to record the actual temperature and humidity in urban areas.

Using Design-Builder energy simulation 5.5 software, compare the amount of heat gained or lost within a structure constructed with conventional bricks (default Scenario used as a reference brick) vs. rice straw–cement bricks and sugarcane bagasse bricks. These types of bio-bricks were chosen because of the availability of these crops in Upper Egypt, in particular. Therefore, the use of its residues will be considered self-sufficiency in the brick industry. to determine the thermal conductivity of bricks with fixed other variables. Note that utilizing the EPW file for the city of Aswan in different scenarios and installing all parameters such as the HVAC system (Fan Coil Unit(4-pipe), Air-cooled Chiller), opening size, and floor and roof layers are required as defined in Table 1^24^.

**Table 1:** Description of different construction elements.

Item	Description	Conductivity value (W/m^2^.k)
External wall	Three layers:	According to several scenarios
	The outermost layer (3 cm cement plaster)	
	Layer 2 (20 cm According to several scenarios)	
	The innermost layer (2 cm gypsum plaster)	
Flat roof	8 Layers:	0.608
	The outermost layer (2 cm concrete tiles)	
	Layer 2 (3 cm cement mortar)	
	Layer 3 (5 cm sand)	
	Layer 4 (7 cm cast concrete)	
	Layer 5 (5 cm M.W. glass wool rolls)	
	Layer 6 (0.4 cm bitumen)	
	Layer 7 (15 cm reinforced concrete)	
	The innermost layer (2 cm cement plaster)	
Internal wall	Three layers:	According to several scenarios
	The outermost layer (2 cm gypsum plaster)	
	Layer 2 (10 cm According to several scenarios)	
	The innermost layer (2 cm gypsum plaster)	
Ground	7 Layers:	1.078
Floor	The outermost layer (Earth)	
	Layer 2 (15 cm cast concrete)	
	Layer 3 (0.4 cm bitumen)	
	Layer 4 (10 cm cast concrete)	
	Layer 5 (6 cm sand)	
	Layer 6 (3 cm cement mortar)	
	The innermost layer (1 cm porcelain)	
Internal	5 Layers:	1.951
Floor	The outermost layer (cement plaster)	
	Layer 2 (15 cm reinforced concrete)	
	Layer 3 (6 cm sand)	
	Layer 4 (3 cm cement mortar)	
	The innermost layer (1 cm porcelain)	

### Simulate default scenarios

**Figure 6:**
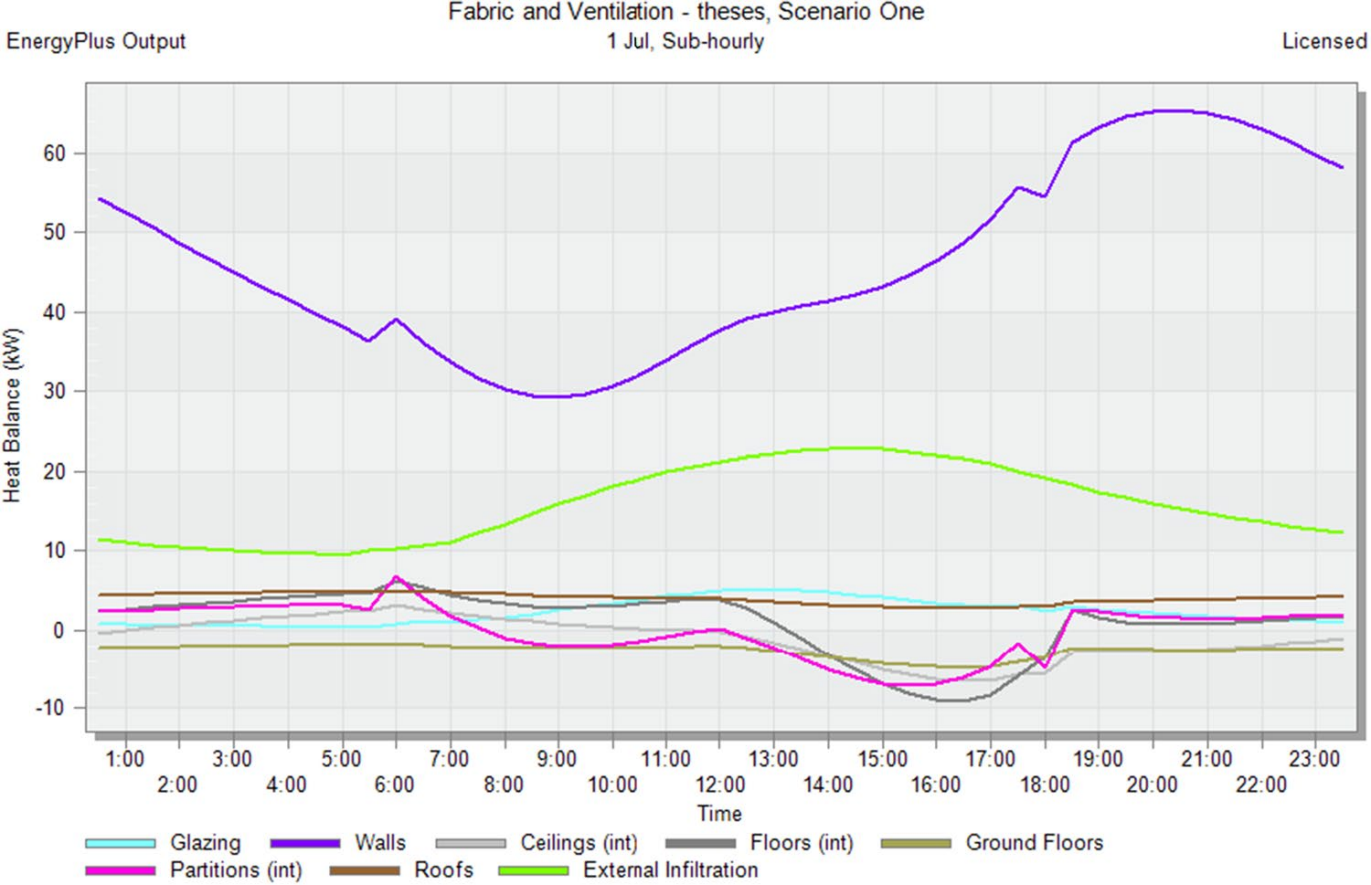
Heat transfer through construction elements.

**Figure 7:**
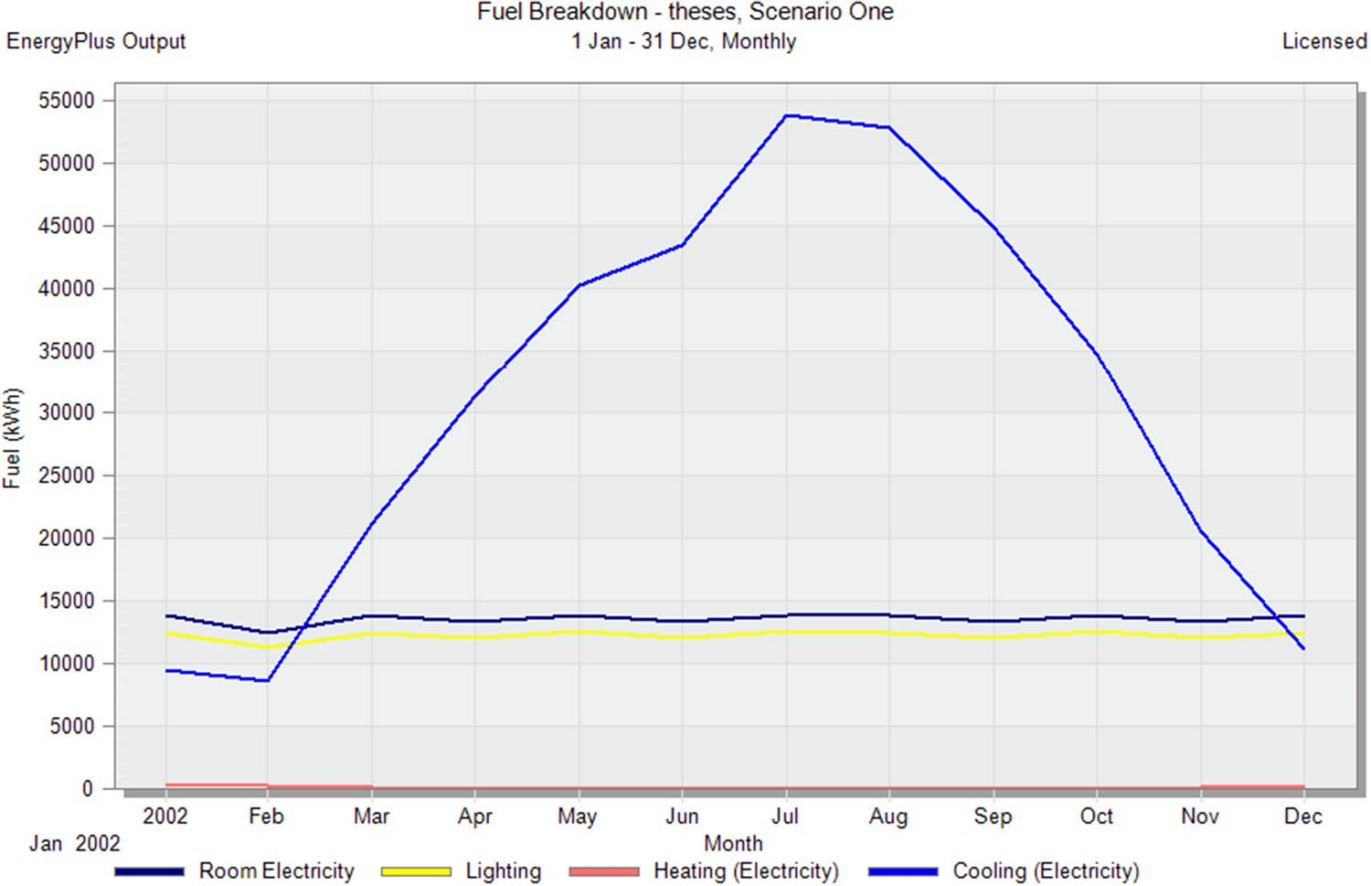
Distribution of electricity in residential building.

**Figure 8:**
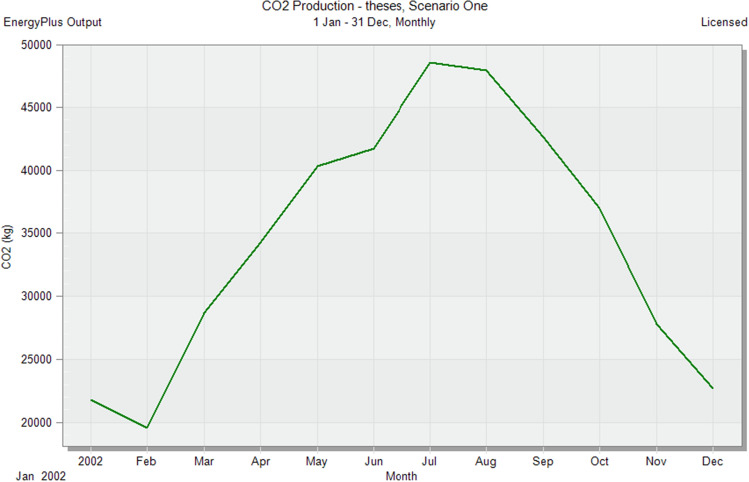
CO_2_ emissions from residential buildings.

In the initial stage of this research, conventional brick (red-fired clay brick) is tested by preparing a model for one of the blocks in the case-study area with the existing material and original orientation to determine the amount of heat gain inside the residential unit through the construction material as shown in Fig. 6. According to the climate consultant software program, thermal comfort for users in Aswan City ranges from 19.5 to 26 °C. So, need to cool them to equal the amount of heat that has been gained through construction materials to achieve thermal comfort and health quality inside residential units. The amount of CO_2_ emitted because of using electricity to cool the building will rise due to this increased need for cooling as shown in Figs. 7 and 8. Bricks play a crucial role in air conditioning. The wall substantially impacts heat transmission and embodied carbon (kgCO_2_), as shown in Table 2^25^.

**Table 2:** Amount of embodied carbon emissions for different building materials.

Materials embodied carbon and inventory	Area (m2)	Embodied Carbon (kgCO_2_)	Mass (kg)
Traditional brick	2547.1	139,291.4	633,142.7
Cement/plaster/mortar–gypsum plaster	3420.3	6846.6	57,055.0
Cement/ plaster/mortar–cement plaster	3468.0	28,791.2	151,532.8
Cement/plaster/mortar–cement mortar	2093.2	19,686.1	103,611.1
Earth common	299.0	4365.7	218,286.1
Painted Oak	104.4	0.0	2557.5
Ceramic/ porcelain	1794.1	26,822.3	41,265.0
Concrete Tiles (roofing)	299.0	2763.0	12,558.9
Sandstone	2093.2	0.0	269,717.8
MW glass wool (rolls)	299.0	274.5	179.4
Concrete reinforced (with 2% steel)	1794.1	200,225.1	645,887.5
Cast concrete	897.1	15,309.9	191,374.1
Bitumen pure	598.0	1205.7	2511.8
Sub total		445,581.5	2,329,679.8

### Simulate rice straw–cement brick scenario

**Figure 9:**
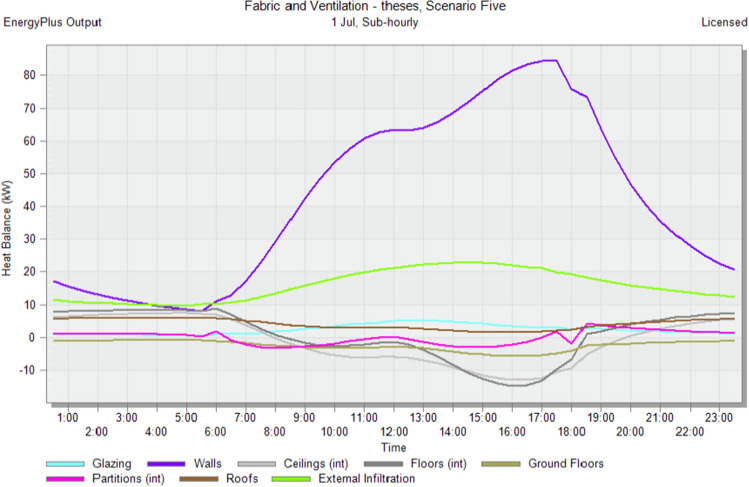
Heat transfer through construction elements after using rice straw–cement brick.

**Figure 10:**
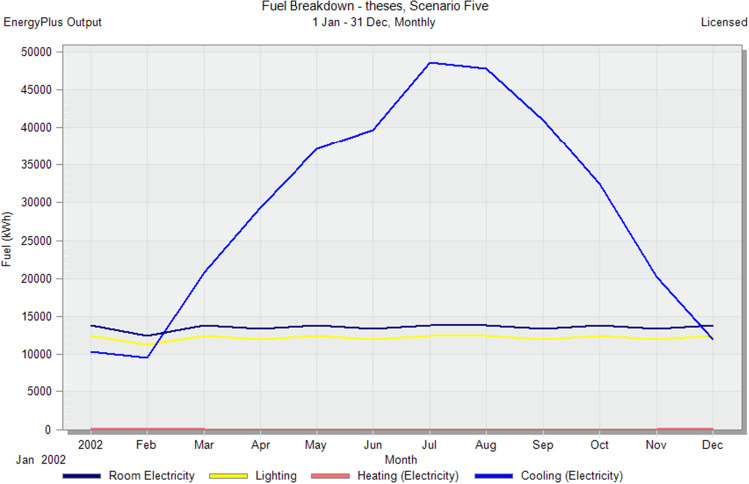
Distribution of electricity in the residential building after using rice straw–cement brick.

**Figure 11:**
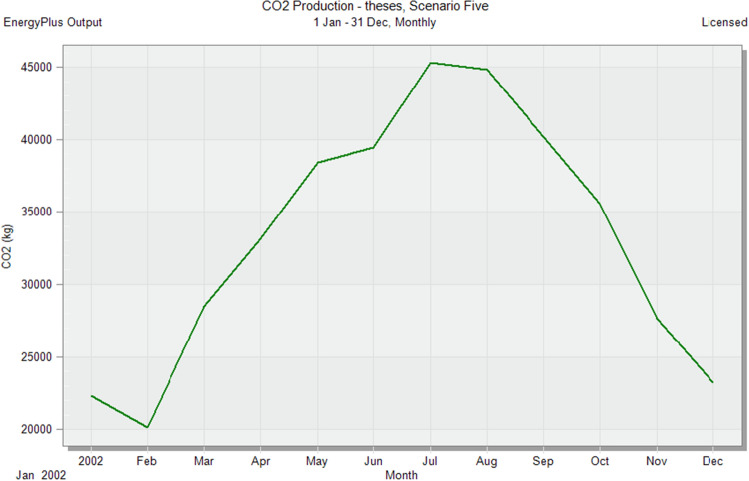
CO_2_ emissions from residential building after using rice straw–cement brick.

In this stage, the outer skin of the structure (Bricks) is improved by using rice plant agriculture waste to generate rice straw–cement bricks with conductivity 0.41 (W/m–k) and density 884 (Kg/m3) ^26^. Figure 9 demonstrates that using rice straw–cement bricks reduce the heat transfer through walls, and the heat balance graph of walls becomes smoother than in the Default Scenario, minimizing the disparity between values, reducing the need to use air-conditioning to cool units, as shown in Fig. 10, and reducing embodied carbon (kgCO_2_) as shown in Fig. 11. On the other side, it will help to enhance the environment by reducing the burning of rice straw, which causes black clouds in Egypt, according to Omayma’s study about agricultural waste, more than 70% of rice straw is not used, and it generates about 3.6 million tons annually^3^.

### Simulate sugarcane bagasse brick scenario

**Figure 12:**
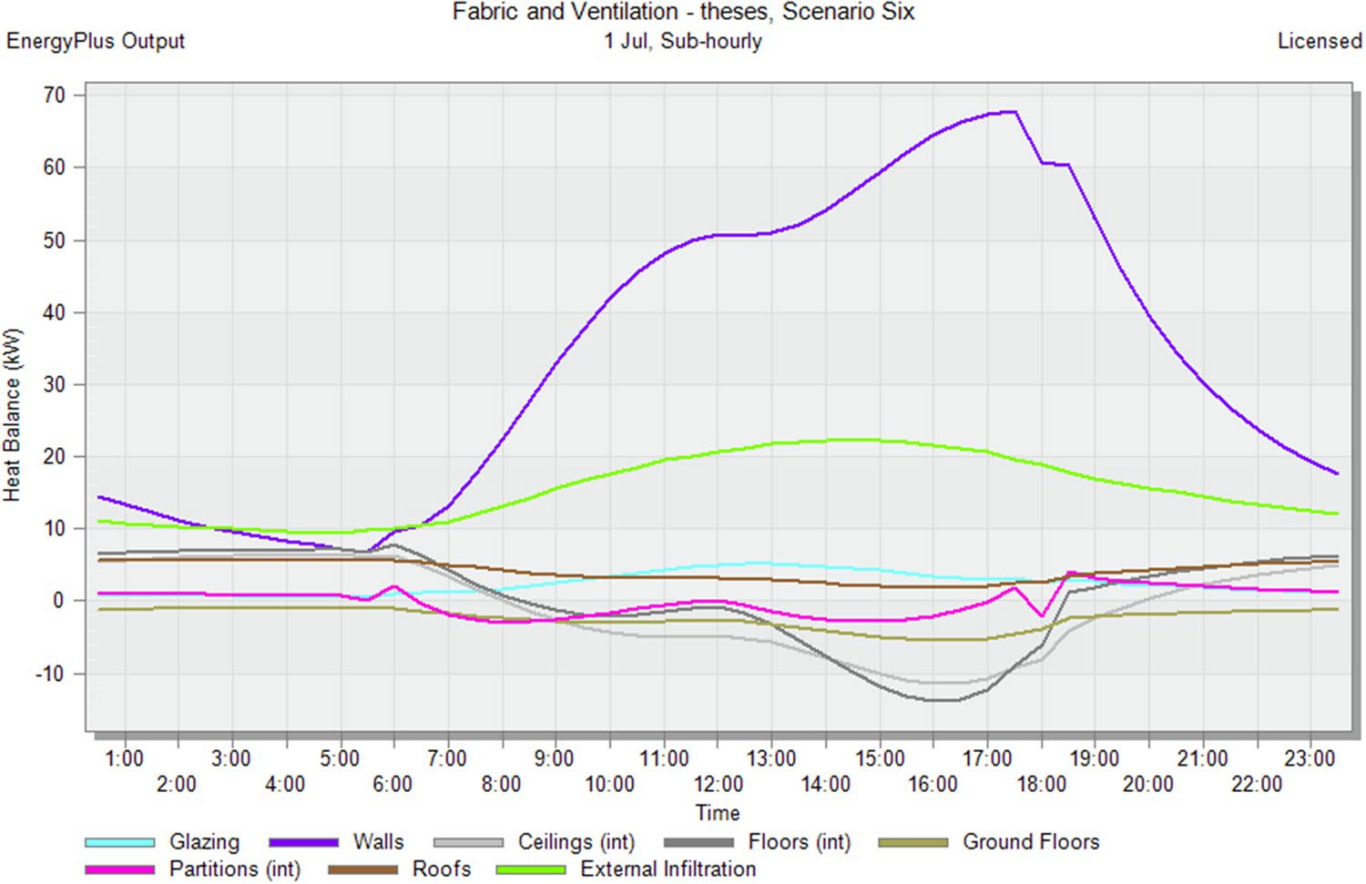
Heat transfer through construction elements after using sugarcane bagasse brick.

**Figure 13:**
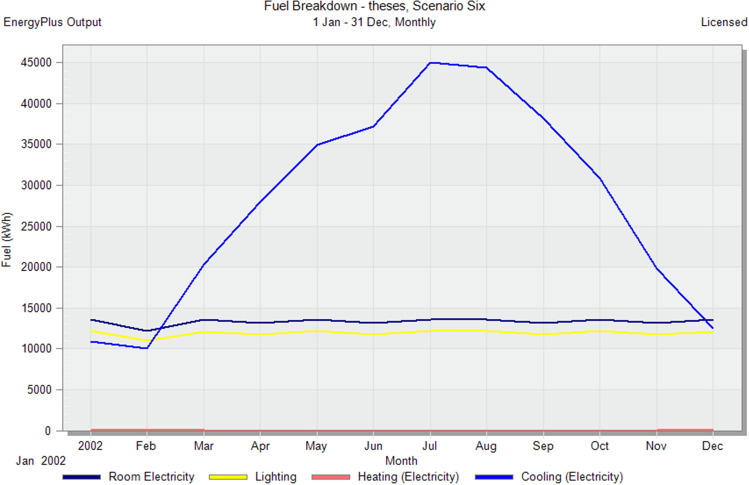
Distribution of electricity in the residential building after using sugarcane bagasse brick.

**Figure 14:**
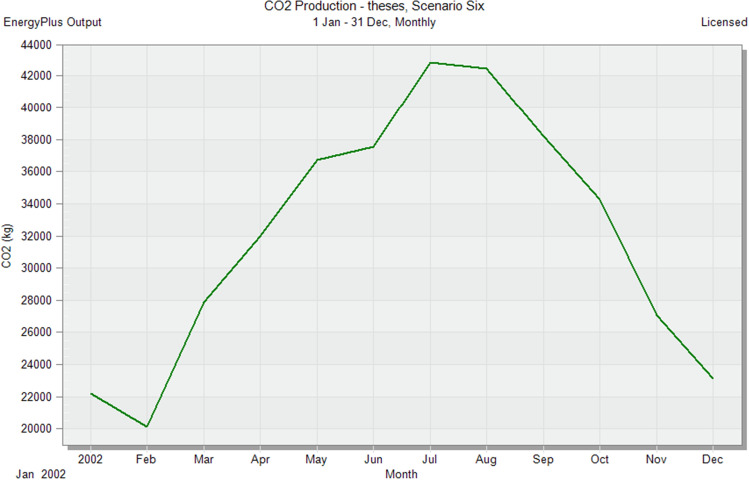
CO_2_ emissions from residential building after using sugarcane bagasse brick.

At this stage, the outer shell of the structure (bricks) is improved using sugarcane plant residues as a result of several previous studies that studied the physical properties of sugarcane^27^ residues to generate sugarcane bricks with a conductivity of 0.27 (W/m–k) and a density of 424 (Kg/m3). Figure 12 demonstrates that using sugarcane bagasse bricks reduces the heat transfer through walls, and the heat balance graph of walls becomes smoother than in the Default Scenario and rice-straw cement brick. and minimizing the disparity between values, reducing the need to use air-conditioning to cool units, as shown in Fig. 13, and reducing embodied carbon (kgCO_2_) as shown in Fig. 14. Egypt generates annually about 5 tons of sugar bagasse cone. But it is an unexploited amount, but the amount of agricultural waste increases annually and is disposed of in non-environmental ways, such as burning it^3^.

## Results and discussion

**Figure 15:**
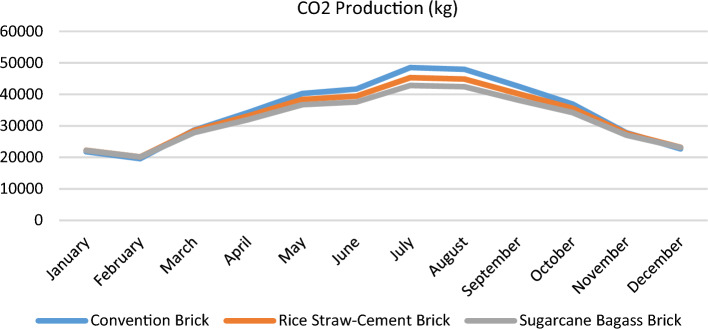
Comparison between different scenarios in CO_2_ Production.

**Figure 16:**
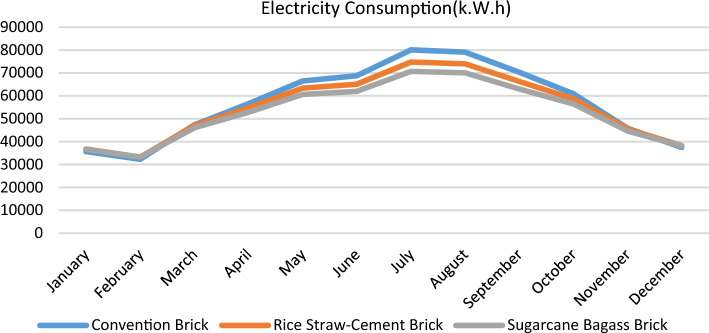
Comparison between different scenarios in electricity consumption.

Based on the Results of the scenarios, which showed how little heat was gained through the walls designed with bio-bricks compared to those designed with traditional bricks. As seen in Fig. 15, increasing the thermal transmittance (U value) of a material will reduce CO_2_ emissions and greenhouse gas (GHG) emissions, and will eliminate the need to cool interior structures. Figure 16 demonstrates that this form of bio-bricks (rice straw–cement bricks & sugarcane bagasse bricks) would reduce the energy consumption of interior buildings, as shown in Table 3. It is also considered an environmental solution for recycling agricultural waste, which Egypt enjoys, and a more economical solution than traditional bricks, because it is less expensive to manufacture than its counterpart and less expensive in the long run (operating).

**Table 3:** Comparison of different scenarios' thermal characteristics.

Item	Conventional brick	Rice straw–cement brick	Sugarcane bagasse brick
Resistivity (m.k/W)	1.18	2.4	3.7
Conductivity (W/m–k)	0.85	0.41	0.27
Density (Kg/m3)	1500	884	423.7
Electricity Consumption in July (kW.H)	80,062	74,772	70,677
CO_2_ Production in July	48,517	45,312	42,830

According to Fig. 16 and Table 4, to calculate the life-cycle assessment of electricity consumption to determine the present value of electricity cost during 20 years for one block. So, assume the cost was calculated according to the inflation rate factor, then return the cost value in year number 20 to the present value to compare the electricity cost for various scenarios and calculate the saving in electricity bills in $ through 20 years as shown in Table 5^28,29^.

**Table 4:** Annual electricity load and cost for different scenarios.

	Convention brick	Rice straw-cement brick	Sugarcane bagasse brick
Annual electricity load (k.w.h)	56,736.33	54,816.85	52,863.15833
Annual electricity cost in $	2658.08	2568.16	2476.63

**Table 5:** The future value of electricity consumption cost for different scenarios.

Period	Convention brick	Rice straw-cement brick	Sugarcane bagasse brick
Year 1	2658.083	2568.156	2476.626
Year 2	3728.227	3602.096	3473.716
Year 3	5229.211	5052.299	4872.234
Year 4	7334.492	7086.355	6833.795
Year 5	10,287.36	9939.322	9585.081
Year 6	14,429.05	13,940.89	13,444.03
Year 7	20,238.18	19,553.5	18,856.6
Year 8	28,386.08	27,425.73	26,448.27
Year 9	39,814.31	38,467.33	37,096.34
Year 10	55,843.55	53,954.28	52,031.33
Year 11	78,326.17	75,676.27	72,979.15
Year 12	109,860.3	106,143.5	102,360.5
Year 13	154,090	148,876.9	143,570.9
Year 14	216,126.7	208,814.8	201,372.6
Year 15	303,139.3	292,883.6	282,445.1
Year 16	425,183.2	410,798.6	396,157.6
Year 17	596,361.9	576,186.1	555,650.6
Year 18	836,457.2	808,158.6	779,355.5
Year 19	1,173,215	1,133,523	1,093,124
Year 20	1,645,551	1,589,880	1,533,216

The financial value equivalent to the cost of electricity consumption for the building was evaluated for a period of 20 years, considering the inflation rate of Egypt in $, which reached 40.26%, based on the Central Bank of Egypt, in order to study the viability of using bio-brick instead of conventional bricks, which resulted in a reduction in electricity consumption in residential buildings according to the following equation $$Future Value=Cost Value*(1+inflation rate\mathrm{ \%})$$.

To calculate the amount of savings in electricity bills, return the future cost value in the previous table to the present value using the following equation $Present Value=\frac{Future Value}{{(1+r)}^{n}}$. As shown in Table 6.

**Table 6:** Precent value of electricity consumption cost during all period and amount of saving in different scenarios.

Period	Convention brick	Rice straw-cement brick	Amount of saving by using rice-straw cement brick	Sugarcane bagasse brick	Amount of saving by using sugarcane bagasse brick
Year 1	2238.386	2162.658	75.728	2085.58	152.806
Year 2	2643.84	2554.395	89.445	2463.355	180.485
Year 3	3122.737	3017.09	105.647	2909.559	213.177
Year 4	3688.379	3563.596	124.784	3436.588	251.791
Year 5	4356.481	4209.094	147.386	4059.081	297.4
Year 6	5145.6	4971.516	174.084	4794.33	351.27
Year 7	6077.657	5872.041	205.616	5662.759	414.898
Year 8	7178.545	6935.684	242.861	6688.494	490.05
Year 9	8478.844	8191.992	286.852	7900.026	578.818
Year 10	10,014.68	9675.863	338.812	9331.012	683.663
Year 11	11,828.7	11,428.52	400.183	11,021.2	807.5
Year 12	13,971.32	13,498.64	472.671	13,017.55	953.767
Year 13	16,502.04	15,943.75	558.289	15,375.51	1,126.53
Year 14	19,491.16	18,831.75	659.416	18,160.58	1,330.586
Year 15	23,021.73	22,242.87	778.860	21,450.13	1,571.604
Year 16	27,191.81	26,271.87	919.941	25,335.53	1856.279
Year 17	32,117.25	31,030.48	1086.576	29,924.73	2192.52
Year 18	37,934.87	36,651.48	1283.394	35,345.2	2589.67
Year 19	44,806.27	43,290.41	1515.864	41,747.52	3058.75
Year 20	52,922.34	51,131.9	1790.443	49,309.54	3612.802

## Conclusion

Structures cause about one-third of greenhouse gas emissions. This is because it symbolizes construction materials (bricks, concrete, tiles, etc.). After all, they need massive amounts of energy in different phases (manufacturing, mobility, construction, and operation), which increases the burden on the environment. These environmental problems increase the effects of climate change, which has become a reality of living. Therefore, all research efforts are focused on finding more environmentally bio-building materials with less harmful effects. So, this study aims to evaluate the findings of earlier laboratory studies to discover new kinds of modified and improved bio-bricks in terms of their thermal qualities. As a result, the results were put to the test by simulating the amount of electrical energy needed to cool the building. Considering that modelling software uses conventional materials to determine overall cooling loads, the use of biomaterials will cut CO_2_ emissions.

- Manufacturing has less impact on the surrounding environment than conventional methods.
- Developing a plan to dispose of agricultural waste without harming the environment.
- To improve the interior environment and human health, provide suggestions for using alternative building materials for more sustainable construction.
- Increasing the thermal transmittance of materials will reduce residential buildings' power use.

To achieve self-sufficiency for the city in the construction sector and to develop new types of essential building materials with thermally improved properties to improve energy efficiency in various types, future studies will point researchers to the possibility of using the various types of waste that are generated in the city of Aswan and the possibility of recycling them in the manufacture of various building materials. structures in places with unique climate features (hot, arid areas) like Aswan.

## Data Availability

The datasets used and/or analysed during the current study are available from the corresponding author on reasonable request.

## References

[1] Melia P, Ruggieri G, Sabbadini S, Dotelli G (2014). Environmental impacts of natural and conventional building materials: A case study on earth plasters. J. Clean. Prod..

[2] Cabeza LF, Rincón L, Vilariño V, Pérez G, Castell A (2014). Life cycle assessment (LCA) and life cycle energy analysis (LCEA) of buildings and the building sector: A review. Renew. Sustain. Energy Rev..

[3] Fouad, Y. in *Egupt's First Biennial Update Report*. (Ministry of Environment, Egyptian Environmental Affairs Agency, Cairo, 2018).

[4] Elbasiouny, H., Elbanna, B.A., Al-Najoli, E., Alsherief, A., Negm, S., Abou El-Nour, E., Nofal, A. & Sharabash, S. Agricultural waste management for climate change mitigation: Some implications to Egypt. In *Waste Management in MENA Regions*, 149–169 (Springer, 2020).

[5] Pezeshki Z, Soleimani A, Darabi A, Mazinani SM (2018). Thermal transport. Build. Mater. Constr. Build. Mater..

[6] Sandak, A., Sandak, J., Brzezicki, M., Kutnar, A., Sandak, A., Sandak, J., Brzezicki, M. & Kutnar, A. Biomaterials for building skins. In *Bio-based Building Skin*, Hong Kong, 27–64 (Springer Open, 2019).

[7] Yadav M, Agarwal M (2021). Biobased building materials for sustainable future: An overview. Mater. Today Proc..

[8] Kadir, A. A., Sarani, N. A., & Leman, A. M. Testing on building material using waste material in fired clay brick. In *Materials Science Forum,* 330–336 (2015).

[9] Farnea, R., Manea, D. L., Tamas-Gavrea, D. R. & Rosca, I. C. Hemp-clay buildingmaterials—An investigation on acoustic, thermal and mechanical properties. In *The 12th International Conference Interdisciplinarity in Engineering*, (Brasov, 2019).

[10] Walker R, Pavía S (2014). Moisture transfer and thermal properties of hemp–lime concretes. Constr. Build. Mater..

[11] Sakhare VV, Ralegaonkar RV (2016). Use of bio-briquette ash for the development of bricks. J. Clean. Prod..

[12] Fernando PR, Krishanth S, Rathnayake NB, Welarahne SA (2019). Manufacturing, physical and chemical characterization of fire clay brick value added with cow dung ash. Am. J. Mater. Synthesis Process..

[13] E. M. a. S. Agency, "Population," Egyptian Mobilization and Statistics Agency (report), 2021.

[14] U. S. E. P. United States Environmental Protection Agency, "Climate Change and Human Health," 7 June 2022. [Online]. Available: https://www.epa.gov/climate-change/climate-change-and-human-health. Accessed jul 2022.

[15] United Nations High Commissioner for Human Rights, U. N. H. C. f. H. Frequently asked questions about human rights and climate change. (Newyork, 2022).

[16] Rajeh, A. Z. Egyptian urbanism: Monitoring developments in the urbanization of Egypt in the late twentieth century and exploring its future paths until 2020. Vol. 2, In *The Historical Development of the Construction Sector in Egypt*, 327-350 (Academic Library, Egypt, 2008).

[17] T. E. &. R. E. Ministry, The Electricity & Renewable Energy Ministry (report) "Annual report of the Electricity Holding Company," Egypt, 2020–2021.

[18] Ministry of Planning & Economic Development M. O. P. &. E. Development, Puplic Investments. May 2022. [Online]. Available: https://mped.gov.eg/Investment?lang=en.

[19] C. A. F. P. M. &. Statistic, "Annual Bulletin of Construction & Bulding Statistics for Public / Pulic Bussines Sector Companies 2019/2018," Egypt, March 2021.

[20] Fatima Ajrama, Hamdi Abdel-Fattah, Maher Al-Nimr. Analysis and evaluation of the risks facing the construction sector in Egypt since the beginning of the nineties. *Eng. Res. J.,* 409–416 (2011).

[21] Omyma Swan, Mahmoud Mostafa, Mohammed Bakry, Shaban Abu hessin, Meshel Farag, Hamdy Mahmoud. Omyma Swan Agricultural Waste Recycling Manual. (Ministry of State for Environmental Affairs, Egypt, 2010).

[22] Geologist, "Row materials for building and construction materials in Egypt," 4 Novamber 2020. [Online]. Available: https://geologist-eg.com/.

[23] Mohamed, A. F. A. & Abdelhady, R. E. Renovation of nile cornish and ancient touristic market in Aswan City; attempt to solve the public transportation problem. In *Cities’ Identities Through its History of Architecture and Arts,* (2020).

[24] Rania Emad Abd El-Hady Ismail. An effect of bio-brick for energy consumption in residential building case study: An existing residential building in Aswan, Egypt M.Sc Thesis, (Arab Academy for Science, Technology & Maritime Transport, South Valley, 2022).

[25] Zayan, A. A., Mohamed, A. F. & Abd El-Hady, R. E. Effect of bio-material in thermal insulation case-study: Energy saving in residential building in Aswan City. In *IOP Conference Series: Earth and Environmental Science,* (2022).

[26] Akmal, T., Fahmy, M., & El-Kadi, A. W. Rice-straw based cement brick microclimatic thermal impact assessment in Cairo, Egypt. In *World Renewable Energy Congress 2011*, (Sweden, 2011).

[27] Adefris Legesse, A., Desalegn, D., Selvaraj, S. K., Paramasivam, V. & Chadha, U. Experimental investigation of sorghum stalk and sugarcane bagasse hybrid composite for particleboard. In *Advances in Materials Science and Engineering,* 1–17 (2022).

[28] Rautray, P., Roy, A., Mathew, D. J., & Eisenbart, B. Bio-brick—Development of sustainable and cost effective building material. In *International Conference on Engineering Design*, India, (2019).

[29] Mohamed AF (2020). Comparative study of traditional and modern building techniques in Siwa Oasis, Egypt: Case study: Affordable residential building using appropriate building technique. Case Stud. Constr. Mater..

